# High Triglycerides Are Associated with Low Thrombocyte Counts and High VEGF in Nephropathia Epidemica

**DOI:** 10.1155/2016/8528270

**Published:** 2016-12-05

**Authors:** Ekaterina V. Martynova, Aygul H. Valiullina, Oleg A. Gusev, Yuriy N. Davidyuk, Ekaterina E. Garanina, Venera G. Shakirova, Ilsiyar Khaertynova, Vladimir A. Anokhin, Albert A. Rizvanov, Svetlana F. Khaiboullina

**Affiliations:** ^1^Institute of Fundamental Medicine and Biology, Kazan Federal University, Kazan, Tatarstan, Russia; ^2^RIKEN, Yokohama, Japan; ^3^Kazan State Medical Academy, Kazan, Tatarstan, Russia; ^4^University of Nevada, Reno, NV, USA

## Abstract

Nephropathia epidemica (NE) is a mild form of hemorrhagic fever with renal syndrome. Several reports have demonstrated a severe alteration in lipoprotein metabolism. However, little is known about changes in circulating lipids in NE. The objectives of this study were to evaluate changes in serum total cholesterol, high density cholesterol (HDCL), and triglycerides. In addition to evaluation of serum cytokine activation associations, changes in lipid profile and cytokine activation were determined for gender, thrombocyte counts, and VEGF. Elevated levels of triglycerides and decreased HDCL were observed in NE, while total cholesterol did not differ from controls. High triglycerides were associated with both the lowest thrombocyte counts and high serum VEGF, as well as a high severity score. Additionally, there were higher levels of triglycerides in male than female NE patients. Low triglycerides were associated with upregulation of IFN-*γ* and IL-12, suggesting activation of Th1 helper cells. Furthermore, levels of IFN-*γ* and IL-12 were increased in patients with lower severity scores, suggesting that a Th1 type immune response is playing protective role in NE. These combined data advance the understanding of NE pathogenesis and indicate a role for high triglycerides in disease severity.

## 1. Introduction

Nephropathia epidemica (NE) is a mild form of hemorrhagic fever with renal syndrome (HFRS), characterized by kidney insufficiency and hemorrhagic disorders. The causative agent is* Puumala virus* (PUUV), member of* Hantavirus* genus, family Bunyaviridae. PUUV targets endothelial cells as viral antigens have been frequently found in endothelial cells, in postmortem tissue [1, 2]. PUUV infection is not cytopathic as cell death attributed to virus replication has not been reported either in vivo or in vitro [1–4]. Therefore the* Hantavirus* pathogenesis remains largely unknown.

NE is endemic in the Republic of Tatarstan where the highest annual prevalence of 64.4 cases per 100,000 was registered in 1997 [5]. Clinically, NE is characterized by an acute onset of high fever, headache, and abdominal pain. Soon, back pain and decrease urine output are followed indicating the impaired renal function. This disease is characterized by 4 periods, febrile, oliguric, diuresis, and convalescence. The oliguric period is the most critical due to the high likelihood of developing life threatening complications. Recovery begins with the onset of diuresis [1, 2]. At this stage, complications can include disseminated encephalomyelitis and hypopituitarism. NE is characterized by a low fatality rate (0.4%). Postconvalescent sequelae are rare and may involve increased glomerular permeability and moderate hypotension [6, 7].

There are 3 clinical forms of NE: mild, moderate, and severe. The severe form of NE is characterized by prominent hemorrhagic symptoms including petechial and nasal and internal bleeding. In some cases, disturbed blood coagulation presents as disseminated intravascular coagulation (DIC) [8]. Laboratory findings for the severe form include high blood urea and creatinine levels, blood urea nitrogen (BUN) >20 mmol/L, and creatinine up to 600 *μ*mol/L. The moderate form of NE has similar but subtler symptoms, with BUN and creatinine levels over 19 mmol/L and 200–300 mmol/L, respectively. The mild form often remains undiagnosed and characterized by mild headache and fever, with hemorrhagic symptoms restricted to small petechia on mucosa and skin.

Increased vascular permeability is a hallmark of NE pathogenesis. Clinically, this increased vascular leakage manifests as petechia, subconjunctival and gastrointestinal hemorrhaging, and, in severe cases, DIC [9, 10]. Additionally, signs of disturbed hemostasis are evident in laboratory tests including prolonged bleeding, increased prothrombin time, and activated partial thromboplastin times [8]. Furthermore, decreased thrombocyte counts are commonly found in NE patients [11, 12]. Extreme thrombocytopenia has been suggested to be predictive of disease severity. Thrombocytopenia (<60 × 10^9^ platelets/L) was found in patients with serum creatinine >620 *μ*mol/L and was an early prognostic marker for acute renal failure [11]. Thrombocytopenia in NE is associated with platelet consumption, due to endothelial cell activation and repair [11, 13]. Recently, a correlation has been shown between thrombocyte counts and serum VEGF levels. Xu et al. demonstrated that thrombocytopenic disorders, characterized by increased platelet destruction, can exhibit increased levels of plasma VEGF [14]. Although serum VEGF has been shown to be upregulated in HFRS, the severe form of* Hantavirus* infection [15, 16], little is known about the association between VEGF and thrombocyte counts in NE cases.

Elevated serum lipase has been documented in some NE patients [17], as well as increased serum cholesterol, total phospholipids, and triglycerides [18], together with upregulated lipid peroxidation [19]. Another study demonstrated low HDCL and total cholesterol, as well as high levels of triglycerides in NE patients [20]. However, little is known about gender differences in lipidemia or any association between serum lipids and cytokine activation in NE.

The objectives of this study were to evaluate changes in serum total cholesterol, HDCL, triglycerides, and cytokine activation in NE patients based on gender, thrombocyte counts, and VEGF and further analyze the association between the severity of the disease and serum lipid, cytokine, VEGF levels, and gender of NE cases. Increased triglycerides were found in NE cases, while total cholesterol levels did not differ significantly between patients and controls. These data indicated that high triglycerides were associated with the lowest thrombocyte counts and high serum VEGF. Furthermore, we found higher triglycerides in male as compared to female NE. Additionally, low triglycerides were associated with upregulation of IFN-*γ* and IL-12, suggesting activation of Th1 helper cells. Patients with lower severity scores had increased IFN-*γ* and IL-12 suggesting that a Th1 type immune response plays protective role in NE.

## 2. Materials and Methods

### 2.1. Patients

Two hundred and twenty-eight NE patients (190 male, 38 female; 38.6 ± 3.1 years) admitted to Republican Clinical Hospital for Infectious Disease named after Agafonov, Republic of Tatarstan, were recruited. Serum from 64 NE patients was collected twice (early (2.1 ± 1.5) and late (7.5 ± 2.3)), while a single serum sample was obtained from 164 patients. Diagnosis of NE was established based on clinical presentation and was serologically confirmed by detection of anti-*Hantavirus* antibodies. In some cases, diagnosis was confirmed using PCR. Serum samples from 56 controls matched for gender, age (32.7 ± 2.3 years), and region were collected. The Institutional Review Board of the Kazan Federal University approved this study and informed consent was obtained from each study subject according to the guidelines approved under this protocol (article 20, Federal Law “Protection of Health Rights of Citizens of Russian Federation” N323- FZ, 11.21.2011).

### 2.2. Multiplex Analysis

Serum cytokine levels were analyzed using Bio-Plex (Bio-Rad, Hercules, CA, USA) multiplex magnetic bead-based antibody detection kits following manufacturer's instructions. Multiplex kits, Bio-Plex Pro Human Cytokine 27-Plex Panel (IL-1*β*, IL-1Ra, IL-2, IL-3, IL-4, IL-5, IL-6, IL-7, IL-8, IL-9, IL-10, IL-12(p70), IL-13, IL-15, IL-17А, CCL2, CCL3, CCL4, CCL5, CCL11, CXCL10, TNF-*α*, G-CSF, GM-CSF, IFN-*γ*, PDGF-BB, and VEGF) and Bio-Plex Human Cytokine 21-Plex Panel (IL-1*α*, IL-2Ra, IL-3, IL-12(p40), IL-16, IL-18, CCL7, CCL27, CXCL1, CXCL9, CXCL12, HGF, IFN- *α*2, LIF, M-CSF, MIF, *β*-NGF, SCF, SCGF-*β*, TNF-*β*, and TRAIL), were used for detection of a total of 48 analytes. Serum aliquots analyzed were 50 *μ*L. A minimum of 50 beads per analyte was acquired. Median fluorescence intensities were collected using Luminex 100 or 200 analyzer (Luminex, Austin, TX, USA). Data collected was analyzed using MasterPlex CT control software and MasterPlex QT analysis software (MiraiBio, San Bruno, CA, USA). Standard curves for each analyte were generated using standards provided by manufacturer.

### 2.3. Serum Lipid Profile

Fasting serum samples were collected early in the morning. Total cholesterol levels were determined using Novochol-200 kit (Vector-Best, Russia) according the manufacturer's instructions. The optical density (OD 520 nm) of the test serum and calibration sample (provided by the manufacturer) was determined using an Infinite M200 PRO analyzer (Tecan, Port Melbourne, VIC, Australia). Cholesterol levels were calculated using the formula: *C* = (*E*/*E*_*k*_)*∗*4.65 (mmol/L), where *C* is concentration of cholesterol; *E* is optical density of tested serum; *E*_*k*_ is optical density of calibration sample; and 4.65 mmol/L is concentration of cholesterol in calibration sample.

Serum triglycerides were determined using the Triglyceride-Novo kit (Vector-Best, Russia) according to manufacturer recommendations. The optic density (520 nm) of serum sample and calibrator (provided by the manufacturer) was determined using Tecan Infinite M200 PRO analyzer (Australia). Triglyceride concentrations were calculated using the formula: *C* = (*E*/*E*_*k*_)*∗*2.29 (mmole/L), where *C* is concentration of triglycerides; *E* is optical density of serum sample; *E*_*k*_ is optical density of calibration sample; and 2.29 mmol/L is concentration of triglycerides in calibration sample.

Serum levels of HDCL were determined using HDCL-Cholesterol-Novo-A kit (Vector-Best, Russia). Briefly, serum sample (3 *μ*L) or calibrator was mixed with reagent 1 (300 *μ*L), incubated for 5 min at 37°C, and used to determine the optic densities *E*_*s*1_ and *E*_cal1_ for the sample and calibrator (provided by the manufacturer), respectively. Then 100 *μ*L of reagent 2 was added and optical densities *E*_*s*2_ and *E*_cal2_ were measured for the sample and calibrator, respectively. The optic density (OD 650 nm) was determined using Tecan Infinite M200 PRO analyzer (Australia). The HDCL level was calculated using formula: *C* = (Δ*E*_*s*_/Δ*E*_cal_)*∗C*_cal_, where *C* is concentration of HDCL; Δ*E*_*s*_ = *E*_*s*2_ − *E*_*s*1_; Δ*E*_cal_ = *E*_cal2_ − *E*_cal1_; *C*_cal_ is 1.08 mM/L which is concentration of HDCL in calibration sample.

The HDCL concentrations were calculated using the formula: *C* = (Δ*E*_*s*_/Δ*E*_cal_)*∗C*_cal_, where *C* is concentration of HDCL; Δ*E*_*s*_ = *E*_*s*2_ − *E*_*s*1_; Δ*E*_cal_ = *E*_cal2_ − *E*_cal1_; *C*_cal_ is 1.08 mmol/L which is concentration of HDCL in calibration sample.

### 2.4. RT-PCR Detection of Puumala virus (PUUV) Transcripts

Total RNA from 100 *μ*L of serum was extracted using TRIzol® reagent (Life Technologies, Carlsbad, CA, USA). cDNA was transcribed using Super Script kit (Life Technologies, Carlsbad, CA, USA) according to the manufacturer's instructions. Two rounds of PCR were conducted. PCR products were sequenced to confirm* Hantavirus* strain. Primers used were 5′-GTGAGAA ACACACCACAATACTATG-3′ forward and 5′-CTCTGCGT CGTTGGAGTCGTTC-3′ reverse, which amplifies the S segment RNA giving a first-round product of 328 bp, and 5′-CGGACACACAAAGGACAGGG-3′ forward and 5′-GACGCAGAGAAACACAAGTATAATA-3′ reverse, which amplified a second-round product of 302 bp.

### 2.5. Statistical Analysis

Statistical analysis was conducted using Minitab software (Minitab, State College, PA, USA); differences between the medians of compared groups were calculated using the Mann-Whitney test for nonparametric data and were considered significant at *P* ≤ 0.05.

The differential abundance of metabolite concentration was calculated using the Subio Platform (Subio Inc., Kagoshima, Japan) with a differentiation expression threshold of 1.5-fold (*t*-test, *P* < 0.05).

## 3. Results

### 3.1. Clinical Presentation

Two hundred and thirty-six NE cases (190 male, 38 female) were recruited for this study (Table 1). The average period of hospitalization was 13.5 ± 2.7 days. NE diagnosis was based on clinical presentation, epidemiological data, and serological confirmation. Additionally, all 228 NE serum samples were analyzed for presence of PUUV RNA. PUUV RNA was detected in the initial serum samples in 49 cases (21.5%). At this time there were decreased platelet counts (159.4 ± 32.1), which, by the end of hospitalization, was restored to control levels (449 ± 40). Bleeding and varying degrees of blood coagulation disturbances were detected in 46 patients (20.1%). Six cases developed DIC (2.5%).

Patients were grouped based on the presence of signs of disturbed hemostasis. Symptoms were evaluated to assign severity points: 0, absence of bleeding (100 cases; 42.4%); 1, few skin petechia (102 cases; 43.2%); 2, multiple skin petechia, scleral hemorrhages, and gastrointestinal bleeding (28 cases; 11.9%); and 3, DIC (6 cases; 2.5%).

Patients were also grouped based on disease severity based on clinical presentation, mild, moderate, or severe forms. Each presentation was assigned severity points: 0, mild (100 cases; 42.4%); 2, moderate (102 cases; 43.2%); and 3, severe (31 cases; 14.4%).

### 3.2. Serum Lipid Profile Analysis

Significant upregulation of triglycerides and HDCL was found in patients at the early stage of the disease (Table 2) compared to controls. In contrast, levels of triglycerides remained significantly upregulated in the late stage of the disease, whereas HDCL were similar to controls. Serum total cholesterol did not change and remained similar to controls.

It has been previously demonstrated that severe* Hantavirus* cases are characterized by a “lipid paradox,” with serum cholesterol lower but triglycerides higher in HFRS cases than controls [21]. Therefore, we sought to determine whether similar serum lipid profiles are found in severe NE. A total of 6 NE patients (5 male, 1 female; age 30.5 ± 0.7 years) were identified as having severe NE based on the diagnosis of DIC (Table 3). Serum total cholesterol and HDCL levels did not differ from controls; however, triglycerides were significantly higher in severe NE cases than controls. These data support Clement et al. observation of the high triglycerides in severe NE cases [21].

### 3.3. Gender Differences in Serum Lipid and Cytokine Activation Profile in NE Patients

When serum lipid profiles were analyzed based on gender, only male cases had significantly higher triglyceride levels than male controls (Table 4). Although upregulated in female NE serum, triglycerides did not differ significantly when compared to female controls. There were no differences between serum level of triglycerides and total cholesterol between male and female NE patients. HDCL levels were significantly lower in female NE compared to female controls, while HDCL was not significantly different in all male patients. Total cholesterol levels did not differ between male and female NE patients as well as between corresponding gender controls.

**Figure 1:**
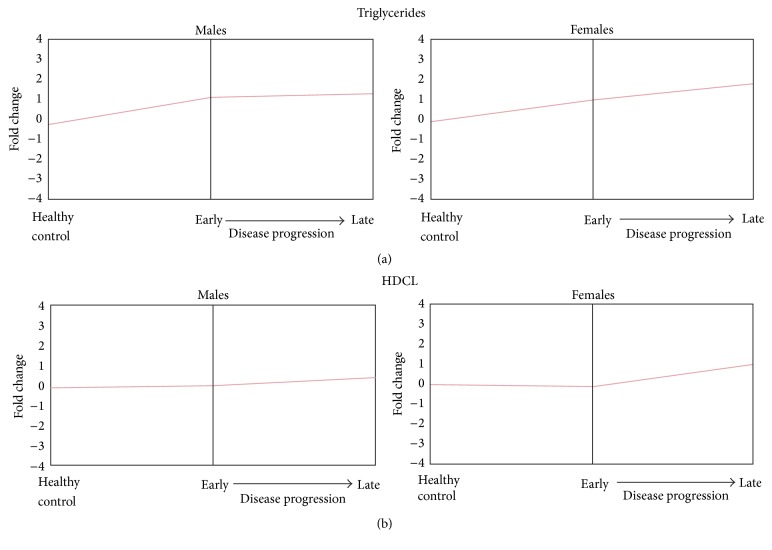
Subio analysis of triglycerides and HDCL in male and female NE cases. Differential abundance of the metabolites concentration was calculated using the Subio Platform (Subio Inc., Kagoshima, Japan) with a differentiation expression threshold of 1.5-fold (*t*-test, *P* < 0.05).

Serum triglyceride levels were significantly increased in NE patients as compared to controls (Figure 1). Also, triglyceride levels differed in NE males and females when compared to controls. It should be noted that once they increased in early stage of the disease, triglycerides remained steady through the convalescent stage in male patients, while female triglycerides continued increasing through early and late stages of the disease (Figure 1(a)). When gender differences in HDCL levels were analyzed, only a slight increase in HDCL level was detected in male NE patients compared to controls, while a steep increase of the HDCL was found in female patients (Figure 1(b)).

Further analysis revealed that more cytokines were significantly upregulated in females than male NE cases when compared to their corresponding controls (33 versus 9) (Table 4). Interestingly, IFN-*γ* and IL-12(p40) were both upregulated in female NE cases, while only IL-12(p40) was significantly higher in male NE cases. Additionally, female cases had significantly lower scores for disease severity (1.9 ± 0.2 versus 2.0 ± 0.1; *P* < 0.05) and hemorrhagic disturbances (0.2 ± 0.1 versus 0.5 ± 0.1; *P* < 0.05) (Table 5). Although not significant, thrombocyte counts in female patients were higher than in males. IFN-*γ* and IL-12 are cytokines that play a key role in the activation of Th1 lymphocytes [22, 23]. Upregulation of these cytokines in female NE suggests that there is activation of Th1 lymphocytes. Previously, we have shown an association between the mild form of NE and elevated serum IFN-*γ* and IL-12 [24]. Therefore, these data suggest that lower severity scores in female cases are associated with activation of Th1 type immune response.

Compared to males, female NE cases had significantly higher levels of TRAIL, CXCL1, and CCL2. These cytokines stimulate mononuclear leukocyte chemotaxis, apoptosis, and angiogenesis. This indicates that differences in clinical presentation between NE in males and females are associated with gender-dependent activation of these cytokines.

### 3.4. Serum Lipid and Cytokine Activation Analysis Based on Thrombocyte Count

Next, changes in serum lipid and cytokines were analyzed based on thrombocyte counts. Thrombocyte counts below 50,000 cells/*μ*L are considered to be a risk factor for bleeding [25–27]. Recently, a correlation was demonstrated between thrombocyte counts and low density lipids in patients with the severe form of dengue infection [28], suggesting a role for lipid metabolism in the pathogenesis of thrombocytopenia. Therefore, we sought to determine whether low thrombocyte counts were associated with changes in lipid and cytokine profiles of NE cases. NE patients were separated into two groups: low (<50,000 cells/*μ*L) and high (>50,000 cells/*μ*L) thrombocyte counts. Disease severity scores did not differ between patients with high and low thrombocyte counts (Table 6). Neither serum total cholesterol nor triglyceride levels differed between these two groups of patients, although serum levels for each lipid were higher than control (Table 7). However, HDCL was significantly increased in NE cases with low thrombocyte counts, while in NE patients with high thrombocyte counts HDCL was significantly decreased.

Analysis of serum cytokines revealed that 30 cytokines were upregulated in patients with low thrombocyte counts, whereas 27 cytokines were upregulated in NE cases with high thrombocyte counts. Overall the activation pattern was similar between groups, with 24 cytokines being upregulated in both groups (Table 7). However, patients with high thrombocyte counts had significantly lower levels of 18 cytokines compared to patients with low thrombocyte counts. One of the most interesting observations was that serum IFN-*γ* and IL-12(p40) were significantly higher in patients with high thrombocyte counts relative to those with low numbers of platelets. One possible explanation of these data is that IFN-*γ* and IL-12(p40) may contribute to thrombocyte protection, preventing hemorrhaging and bleeding. Additionally, significantly higher levels of IL-3 and IFN*α* were found in patients with high thrombocyte counts. Furthermore, serum levels of IFN-*α* differed significantly between the two groups of patients with different thrombocyte counts. Since the activity of IFN-*α* and IL-3 overlaps, targeting dendritic cell proliferation (IL-3) and innate protection (IFN-*α*), this suggests that activation of dendritic cells may play a role in preventing the drop of thrombocyte counts in NE.

### 3.5. Analysis of Lipid and Cytokine Serum Profile in NE Patients with High or Low VEGF Levels

Studies have shown a correlation between VEGF serum levels and the concentrations of cholesterol and triglycerides [29, 30], with high triglycerides and cholesterol associated with elevated VEGF, presumably due to endothelial damage. Therefore, we sought to determine whether lipid profiles differed in patients with high or low serum VEGF. NE patients were divided into two groups, with the highest control VEGF concentration (93 pg/mL) used as a separation marker (Table 8). The NE group with low VEGF levels was characterized by significantly lower thrombocyte counts as compared to NE cases with high VEGF. Furthermore, the low VEGF group had higher bleeding severity scores relative to the high VEGF group, although differences were not statistically significant. Interestingly, all 6 DIC cases were grouped in the low VEGF category, suggesting that low VEGF is associated with a higher chance of developing severe bleeding. Both NE groups had high serum triglycerides compared to controls; however, significantly increased concentrations of cholesterol and triglycerides were found in the high VEGF group relative to the low VEGF group. Although HDCL did not differ from that in control in either NE group, they differed significantly between the NE groups, with significantly lower HDCL in the high VEGF group.

Cytokine analyses revealed more cytokines upregulated (32 out of 48) in the high VEGF group than the low VEGF group (26 out of 48) (Table 9). Although the overall pattern of cytokine activation was similar between the two groups, 26 cytokines had significant upregulation in the high VEGF group when compared to the low VEGF group. It appears that patients with high VEGF levels have more pronounced activation of Th1 cytokines, since elevated IFN-*γ*, a cytokine produced by Th1 lymphocytes, was characteristic of the group. Though serum levels of IL-12(p40) were similar between two groups of patients, high VEGF patients had significantly higher levels of IL-12(p70), a IL-12(p40) precursor.

## 4. Discussion

Serum triglycerides were elevated in NE cases. These data support previous observations of Mustonen et al., who demonstrated that high triglyceride concentrations were present in NE serum [20]. Interestingly, serum levels of cholesterol remained unchanged in the NE cases enrolled in our study, in contrast to other studies, where cholesterol was lower in NE as compared to controls [20]. Our data suggest that there is an association between increased triglycerides and severe clinical presentation of NE. This observation supports the conclusions of Clement et al., who demonstrated increased fasting triglyceride concentrations in* Hantavirus* patients [21] and further that increased levels of triglycerides were more pronounced in severe cases. Our data confirm high triglyceride concentrations in severe NE cases relative to controls. We have demonstrated that triglycerides were significantly increased in male patients, who also had higher severity scores as compared to female NE patients. In fact, out of 6 patients diagnosed with severe complications, that is, having DIC, 5 were male. However, unlike the observations of Clement et al., in this study serum cholesterol remained unchanged in NE patients compared to controls. It remains to be determined whether these discrepancies in cholesterol levels are related to regional or national differences in serum lipid level.

We have demonstrated that increased serum IFN-*γ* and IL-12 are associated with the mild form of NE [24]. These data confirm this observation, with a lower NE severity score being associated with higher levels of these cytokines. It should be noted that triglycerides were lower in patients with the severe form of the disease. Although little is known about triglycerides and cytokine activation, it is generally accepted that hyperlipidemia is associated with activation of proinflammatory cytokines, IL-6 in particular. For example, Göçmen et al. have demonstrated that hyperlipidemia is associated with increased serum IL-6, and furthermore, increases in IL-6 were found in obesity characterized by dyslipidemia [31]. A potential mechanism connecting triglycerides and IL-6 was proposed by Valdearcos et al. [31, 32]. These authors demonstrated that lipin-2 may be involved in regulation of triglyceride concentrations and activation of the proinflammatory cytokines TNF-*α* and IL-6. Depletion of lipin-2 increased expression of the IL-6 and TNF-*α*, while its overexpression reduced the release of proinflammatory cytokines. Interestingly, cytokine activation was associated with the ability of lipin-2 to modulate the cellular content of triglyceride, with upregulation of cytokines being associated with high lipid content.

Interestingly, we found significantly higher levels of IL-6 in patients with increased triglycerides in NE cases with higher severity scores. Several studies have shown that the severe form of NE is associated with elevated IL-6 [33–35]. Since IL-6 upregulation could be associated with high serum triglycerides, it could be suggested that metabolic syndrome in NE cases may contribute to proinflammatory cytokine activation, particularly IL-6. High IL-6 could alter lymphocyte differentiation, since IL-6 is known to skew T cell differentiation towards TH2 and TH17 [36–38]. Promoting lymphocyte proliferation in more pathogenic Th17 helpers may have a devastating impact on tissue integrity and explain NE pathogenesis. If this is the case, our data on upregulation of IFN-*γ* and IL-12 in mild NE cases suggests a protective role for Th1 lymphocytes in NE.

Thrombocyte counts are a predictive marker for bleeding [39, 40]. NE is characterized by low thrombocyte counts, which present clinically with petechia, scleral bleeding, gastrointestinal bleeding, or DIC. Since we found no association between triglyceride concentration and thrombocyte counts, this suggests that hypertriglyceridemia and thrombocytopenia are independent events. However, we found an association between high thrombocyte counts and upregulation of the Th1 type cytokines, IFN-*γ* and IL-12(p40). Therefore, we suggest that activation of Th1 lymphocytes is protective in NE patients, interfering with thrombocytopenia. Supporting this hypothesis the data of Diehl and Rincón [38] demonstrated a protective role of the Th1 response, with IFN-*γ* or IL-12 deficient mice being highly susceptible to dengue infection [41]. IFN-*γ* ^−/−^ and IL-12^−/−^ animals developed a lethal infection, which was characterized by severe thrombocytopenia. The mechanism of IFN-*γ* and IL-12 protective roles remains to be investigated.

Significantly higher serum VEGF levels were found in NE patients with increased concentrations of triglycerides. Increased VEGF levels have been demonstrated in hypercholesterolemic patients and in animals fed a high fat diet [29, 30]. Importantly, our data is the first to show an association between hypertriglyceridemia and increased VEGF serum level in NE cases. VEGF can play dual role in regulating endothelial integrity. For example, VEGF can be protective, promoting endothelial cell survival [30, 42]. However, VEGF can also increase endothelial permeability and promote vascular leakage [43]. Studies using animals fed a high fat diet suggest that different VEGF family members vary in their association with hyperlipidemia. It has been demonstrated that VEGF-C, but not VEGF-A, is closely connected to dyslipidemia in animals [30]. Therefore, this suggests that NE cases with high and low triglycerides may differ in levels of different VEGFs. Future studies will determine whether NE cases vary in the upregulation of different VEGFs and how their expression affects disease progression and triglyceride concentrations.

In conclusion, our data confirms previous observation of increased triglycerides in NE cases. This study advances our understanding of the role of triglycerides in NE pathogenesis by demonstrating an association between high triglycerides and patient gender, severity score of the disease, thrombocyte counts, and serum VEGF level. Additionally, our data suggest that low triglycerides are associated with upregulation of Th1 helper cells.

## Figures and Tables

**Table 1:** Clinical characteristics of NE cases.

Variables	Value
Antibody titer (1st)	885 ± 115
Antibody titer (2nd)	2163.5 ± 263
Hospitalization (day)	13.5 ± 2.7
Serum urea (mmol/L)	11.6 ± 0.8
Serum creatinine (*μ*mol/L)	148.8 ± 5.4
Thrombocytes (1st) (×1000 cells/*μ*L)	105.1 ± 4.2
Thrombocytes (2nd) (×1000 cells/*μ*L)	466.9 ± 29.9
Bleeding (number of patients)	46 out of 236
Sex (M/F)	198/38

**Table 2:** Lipid profile in NE patients.

Analyte	Control	NE early stage	NE late stage	*P*
HDCL (mmol/L)	1.6 ± 0.1	1.2 ± 0.1	1.8 ± 0.9	^*∗*^0.05
Total cholesterol (mmol/L)	3.6 ± 0.2	4.1 ± 0.5	4.2 ± 0.6	
Triglycerides (mmol/L)	1.7 ± 0.2	3.5 ± 0.1	3.5 ± 0.3	^*∗*^0.0001; ^*∗∗*^0.0001

^*∗*^ *P* differences are between NE 1st and control.

^*∗∗*^ *P* differences between NE 2nd and control.

**Table 3:** Lipids analysis in NE cases with high bleeding score.

Analyte	Control	NE	*P*
HDCL (mmol/L)	1.6 ± 0.1	1.8 ± 0.6	
Total cholesterol (mmol/L)	3.6 ± 0.2	3.1 ± 0.3	
Triglycerides (mmol/L)	1.7 ± 0.2	2.8 ± 0.4	0.05

**Table 4:** Gender based lipid (mmol/L) and cytokine (pg/mL) analysis.

Analyte	Control female (*n* = 15)	NE female (*n* = 38)	Control male (*n* = 30)	NE male (*n* = 190)	*P*
HDCL	1.7 ± 0.2	1.2 ± 0.1	1.2 ± 0.2	1.2 ± 0.1	^*∗∗*^0.001
Total cholesterol	3.5 ± 0.2	4.5 ± 0.5	3.9 ± 0.2	4.2 ± 0.1	
Triglycerides	1.7 ± 0.2	3.3 ± 0.5	1.6 ± 0.5	3.6 ± 0.2	^*∗*^0.0001

IL-1Ra	8.0 ± 2.9	68.5 ± 13.7	68.3 ± 13.5	126.5 ± 26.6	^*∗∗*^0.001
IL-2Ra	32.9 ± 4.9	101.6 ± 20.4	1.0 ± 0.2	120.1 ± 26.3	^*∗∗*^0.0001
IL-3	74.1 ± 4.5	137.5 ± 20.7	45.9 ± 12.6	124.4 ± 10.9	^*∗∗*^0.0001; ^*∗*^0.05
IL-4	0.8 ± 0.1	13.2 ± 4.5	1.5 ± 0.2	13.2 ± 1.9	^*∗∗*^0.001
IL-5	0.4 ± 0.1	4.0 ± 1.0	1.2 ± 0.2	5.9 ± 0.9	^*∗∗*^0.05
IL-6	1.4 ± 0.3	35.2 ± 0.9	3.7 ± 0.7	34.2 ± 8.4	^*∗∗*^0.01
IL-7	1.7 ± 0.6	7.4 ± 2.1	2.1 ± 0.4	12.8 ± 2.8	^*∗∗*^0.05
IL-8	10.8 ± 2.8	31.1 ± 6.9	44.3 ± 13.9	45.8 ± 9.0	^*∗∗*^0.001
IL-9	2.5 ± 0.4	46.9 ± 15.0	4.5 ± 1.4	64.9 ± 16.8	^*∗∗*^0.05
IL-10	2.6 ± 1.1	22.4 ± 4.4	4.8 ± 0.9	39.6 ± 6.2	^*∗∗*^0.001
IL-12(p40)	110.8 ± 13.8	227.3 ± 43.7	68.0 ± 34.6	186.8 ± 16.2	^*∗∗*^0.01; ^*∗*^0.05
IL-12(p70)	5.4 ± 0.7	25.2 ± 8.4	2.0 ± 0.5	29.1 ± 3.9	^*∗∗*^0.05; ^*∗*^0.05
IL-13	1.4 ± 0.2	23.5 ± 7.8	1.5 ± 0.2	23.8 ± 4.1	^*∗∗*^0.05
IL-15	4.0 ± 0.6	32.5 ± 12.7	8.6 ± 2.7	33.7 ± 9.4	^*∗∗*^0.05
IL-17	2.8 ± 1.0	34.7 ± 20.5	1.7 ± 0.4	25.8 ± 4.9	
IL-18	12.4 ± 2.4	25.2 ± 7.2	5.7 ± 1.9	21.9 ± 2.5	^*∗∗*^0.0001
CCL2	12.2 ± 1.5	124.1 ± 88.7	16.9 ± 3.6	40.9 ± 4.0	^&^0.05
CCL4	4.8 ± 0.8	571.6 ± 188.5	18.6 ± 6.7	685.0 ± 109.8	^*∗∗*^0.5
CCL5	33.1 ± 3.0	2140.8 ± 720.1	86.8 ± 21.6	2288.8 ± 316.9	^*∗∗*^0.01
CCL11	6.4 ± 1.3	67.8 ± 23.1	19.1 ± 4.3	64.3 ± 6.7	^*∗∗*^0.5
CCL27	170.9 ± 11.0	74.3 ± 11.7	72.8 ± 11.4	59.1 ± 5.4	^*∗∗*^0.0001
CXCL1	54.6 ± 10.3	67.2 ± 29.2	11.5 ± 3.3	23.5 ± 5.4	^*∗∗*^0.01; ^&^0.01
CXCL9	131.9 ± 25.2	1646.9 ± 776.2	80.0 ± 18.6	1251.3 ± 124.6	
CXCL10	25.8 ± 3.3	3318.7 ± 806.2	60.4 ± 15.9	2520.8 ± 295.6	^*∗∗*^0.001; ^*∗*^0.01
G-CSF	8.2 ± 1.6	19.6 ± 2.6	8.3 ± 1.0	28.7 ± 2.6	^*∗∗*^0.001; ^*∗*^0.01
HGF	126.2 ± 19.6	311.6 ± 83.9	64.1 ± 24.1	334.4 ± 33.8	^*∗∗*^0.01; ^*∗*^0.05
IFN-*α*	10.7 ± 0.8	25.1 ± 8.6	6.3 ± 2.3	18.2 ± 2.8	^*∗∗*^0.0001
IFN-*γ*	12.9 ± 2.8	53.0 ± 7.6	57.4 ± 10.3	78.8 ± 8.3	^*∗∗*^0.001
MIF	152.2 ± 29.7	262.2 ± 98.6	144.3 ± 63.5	218.4 ± 19.6	^*∗∗*^0.05
PDGF	75.9 ± 11.8	1556.2 ± 453.9	213.9 ± 55.1	5871.4 ± 4204.5	^*∗∗*^0.001
SCGF-bb	2171.1 ± 405.2	3577.2 ± 532.4	678.8 ± 137.2	5139.9 ± 471.0	^*∗∗*^0.0001; ^*∗*^0.001
SDFa	78.2 ± 4.5	74.5 ± 16.5	19.8 ± 6.2	84.8 ± 17.4	^*∗∗*^0.0001
TRAIL	19.1 ± 3.6	51.6 ± 11.4	12.9 ± 5.4	33.4 ± 2.6	^*∗∗*^0.0001; ^*∗*^0.05; ^&^0.05
VEGF	11.8 ± 1.8	103,2 ± 35.1	22.2 ± 7.5	163.8 ± 28.2	^*∗∗*^0.05

^*∗∗*^ *P* value between female control and female NE; ^*∗*^ *P* value between male control and male NE; ^&^ *P* value between male and female NE.

**Table 5:** Clinical presentation of NE patients based on gender.

Criteria	NE female (*n* = 38)	NE male (*n* = 190)	*P*
Severity score	1.9 ± 0.02	2.0 ± 0.1	<0.05
Hemorrhagic syndrome score	0.2 ± 0.1	0.5 ± 0.1	<0.05
Thrombocytes (×1000 cells/*μ*L)	125.7 ± 6.7	101.2 ± 6.0	<0.1

**Table 6:** Clinical presentation of NE patients with high (>50,000) and low (<50,000) thrombocyte counts.

Clinical feature	Low thrombocytes	High thrombocytes	*P*
Severity score	2.1 ± 0.1	2.0 ± 0.01	
Hemorrhagic syndrome score	0.5 ± 0.1	0.5 ± 0.1	
Thrombocyte counts (×1000 cells/*μ*L)	46.5 ± 1.6	123.5 ± 7.4	<0.0001

**Table 7:** Lipids (mmol/L) and cytokine (pg/mL) analysis based on thrombocytes (×1000 cells/*μ*L) counts (low < 50.000 versus high > 50.000).

Analyte	Control (*n* = 56)	Thrombocyte low (*n* = 58)	Thrombocyte high (*n* = 180)	*P*
HDCL	1.6 ± 0.1	1.9 ± 0.1	1.1 ± 0.1	^*∗*^0.05; ^*∗∗*^0.05
Total cholesterol	3.6 ± 0.1	4.1 ± 0.2	4.1 ± 0.2	^*∗*^0.05
Triglyceride	1.7 ± 0.2	3.7 ± 0.4	3.4 ± 0.2	^*∗*^0.05; ^*∗∗*^0.0001

IL-1a	1.3 ± 0.1	0.1 ± 0.03	0.2 ± 0.03	^*∗*^0.01; ^*∗∗*^0.0001
IL-1Ra	29.3 ± 3.1	94.4 ± 14.8	87.7 ± 10.9	^*∗*^0.01; ^*∗∗*^0.01
IL-1b	0.8 ± 0.1	25.6 ± 5.0	12.2 ± 2.7	^*∗∗*^0.01; ^&^0.05
IL-2	1.8 ± 0.3	29.8 ± 9.6	23.11 ± 10.9	^*∗*^0.01
IL-3	66.6 ± 5.8	100.5 ± 15.6	117.4 ± 13.4	^*∗*^0.05; ^*∗∗*^0.05
IL-4	1.1 ± 0.1	30.6 ± 6.5	12.7 ± 2.3	^*∗*^0.01; ^*∗∗*^0.01; ^&^0.01
IL-5	1.0 ± 0.2	12.5 ± 3.0	6.0 ± 0.9	^*∗*^0.01; ^*∗∗*^0.001; ^&^0.01
IL-6	2.9 ± 0.6	47.1 ± 13.5	37.5 ± 12.5	^*∗*^0.01
IL-8	37.9 ± 14.9	102.9 ± 18.7	32.5 ± 4.5	^&^0.001
IL-9	3.5 ± 0.5	105.9 ± 18.8	77.5 ± 25.9	^*∗*^0.001
IL-10	3.6 ± 0.6	68.7 ± 12.1	35.7 ± 7.9	^*∗*^0.001; ^*∗∗*^0.05; ^&^0.05
IL-12(p40)	88.1 ± 12.1	163.0 ± 23.7	185.8 ± 20.3	^*∗*^0.05; ^*∗∗*^0.01
IL-12(p70)	3.7 ± 0.5	54.2 ± 12.9	27.4 ± 4.2	^*∗*^0.01; ^*∗∗*^0.001; ^&^0.05
IL-13	1.3 ± 0.1	42.9 ± 10.2	20.1 ± 3.4	^*∗*^0.001; ^*∗∗*^0.001; ^&^0.01
IL-15	5.4 ± 0.8	49.7 ± 12.8	38.7 ± 14.5	^*∗*^0.01
IL-17	2.3 ± 0.5	46.6 ± 7.9	20.7 ± 3.4	^*∗*^0.001; ^*∗∗*^0.01; ^&^0.001
IL-18	8.6 ± 1.4	^&^19.7 ± 4.0	15.9 ± 2.6	
CCL2	13.0 ± 1.3	60.4 ± 9.2	66.8 ± 28.3	^*∗*^0.01
CCL3	0.9 ± 0.3	94.3 ± 23.8	31.2 ± 6.9	^*∗*^0.001; ^*∗∗*^0.01; ^&^0.001
CCL4	10.3 ± 2.0	1946.8 ± 396.7	634.1 ± 108.6	^*∗*^0.001; ^*∗∗*^0.001; ^&^0.0001
CCL5	60.9 ± 8.4	5078.9 ± 910.5	2484.4 ± 422.1	^*∗*^0.001; ^*∗∗*^0.001; ^&^0.01
CCL11	15.5 ± 2.8	117.3 ± 19.9	55.9 ± 7.8	^*∗*^0.001; ^*∗∗*^0.001; ^&^0.001
CCL27	122.7 ± 9.3	^&^32.9 ± 7.3	55.5 ± 6.1	^*∗∗*^0.0001
CXCL9	126.3 ± 18.2	^&^1720.1 ± 333.7	1334.3 ± 270.4	^*∗*^0.001; ^*∗∗*^0.01
CXCL10	49.1 ± 6.9	5912.8 ± 929.9	2797.1 ± 381.1	^*∗*^0.001; ^*∗∗*^0.0001; ^&^0.001
G-CSF	8.1 ± 0.9	37.6 ± 4.7	23.6 ± 2.1	^*∗*^0.01; ^*∗∗*^0.0001; ^&^0.01
GM-CSF	2.6 ± 0.7	34.6 ± 6.7	16.5 ± 3.7	^*∗*^0.01; ^*∗∗*^0.05; ^&^0.05
HGF	95.9 ± 13.1	455.6 ± 122.6	325.3 ± 38.5	^*∗*^0.01; ^*∗∗*^0.001
IFN-*α*	15.2 ± 1.2	^&^12.8 ± 1.7	20.3 ± 1.5	^*∗∗*^0.01; ^&^0.01
IFN-*γ*	32.6 ± 4.1	62.9 ± 8.3	63.8 ± 5.4	^*∗*^0.05; ^*∗∗*^0.001
MIF	145.7 ± 24.6	^&^235.4 ± 28.6	257.4 ± 38.5	^*∗*^0.05
PDGF	144.5 ± 22.8	3282.7 ± 500.3	1644.0 ± 230.3	^*∗*^0.001; ^*∗∗*^0.0001; ^&^0.01
SCGF	1569.1 ± 234.5	^&^6499.8 ± 939.4	5149.5 ± 617.8	^*∗*^0.05; ^*∗∗*^0.001
TRAIL	15.8 ± 2.4	^&^33.4 ± 5.2	38.7 ± 4.5	^*∗*^0.01; ^*∗∗*^0.01
VEGF	15.2 ± 2.3	249.0 ± 59.6	107.8 ± 18.0	^*∗*^0.01; ^*∗∗*^0.01; ^&^0.01

^&^ *P* between groups.

^*∗*^ *P* between thrombocyte counts < 50 patients and control.

^*∗∗*^ *P* between thrombocyte counts > 50 patients and control.

**Table 8:** Severity score based on serum VEGF (high > 93 pg/mL; low < 93 pg/mL).

Analyte	NE low VEGF	NE high VEGF	*P*
Severity score	2.0 ± 0.01	2.1 ± 0.04	<0.05
Hemorrhagic syndrome score	0.5 ± 0.06	0.3 ± 0.05	
Thrombocytes (×1000 cells/*μ*L)	118.7 ± 6.5	226.6 ± 9.0	<0.01

**Table 9:** Lipids (mmol/L) and cytokine (pg/mL) analysis based on high (>93 pg/mL) or low (<93 pg/mL) serum VEGF level.

Analyte	Control	VEGF high	VEGF low	*P* (between groups)
HDCL	1.6 ± 0.1	0.9 ± 0.3	1.3 ± 0.1	^*∗*^0.001
Total cholesterol	3.6 ± 0.1	4.7 ± 0.3	3.9 ± 0.1	^*∗*^0.01
Triglycerides	1.7 ± 0.2	^&^3.9 ± 0.3	^&^3.2 ± 0.1	^*∗*^0.05

IL-1b	0.8 ± 0.1	^&^39.2 ± 5.2	2.9 ± 0.6	^*∗*^0.0001
IL-1Ra	31.4 ± 5.5	^&^296.3 ± 83.3	^&^87.6 ± 12.5	^*∗*^0.001
IL-2	1.8 ± 0.3	^&^56.2 ± 19.1	4.2 ± 0.6	^*∗*^0.0001
IL-2Ra	29.3 ± 3.2	^&^203.1 ± 73.8	^&^101.7 ± 9.2	^*∗*^0.05
IL-3	66.6 ± 6.0	^&^166.8 ± 38.6	^&^154.7 ± 15.9	
IL-4	1.1 ± 0.1	^&^36.9 ± 4.5	2.7 ± 0.4	^*∗*^0.0001
IL-5	1.0 ± 0.2	^&^13.8 ± 2.1	^&^2.3 ± 0.2	^*∗*^0.0001
IL-6	2.9 ± 0.7	^&^96.5 ± 22.6	^&^11.0 ± 1.1	^*∗*^0.0001
IL-7	2.1 ± 0.4	^&^31.6 ± 7.2	^&^3.93 ± 0.5	^*∗*^0.0001
IL-8	37.9 ± 15.7	^&^100.7 ± 23.3	24.0 ± 2.4	^*∗*^0.0001
IL-9	3.5 ± 0.5	^&^173.7 ± 43.8	^&^19.5 ± 3.0	^*∗*^0.0001
IL-10	3.6 ± 0.7	^&^99.2 ± 21.5	^&^20.7 ± 3.9	^*∗*^0.0001
IL-12(p40)	88.1 ± 12.7	^&^233.2 ± 41.6	^&^228.5 ± 17.6	
IL-12(p70)	3.7 ± 0.5	^&^75.7 ± 9.4	9.5 ± 1.8	^*∗*^0.0001
IL-13	1.3 ± 0.1	^&^6.5 ± 10.2	^&^6.6 ± 0.7	^*∗*^0.0001
IL-15	5.4 ± 0.9	^&^91.8 ± 25.2	^&^10.9 ± 1.2	^*∗*^0.0001
IL-17	2.3 ± 0.6	^&^73.9 ± 15.4	6.4 ± 1.8	^*∗*^0.0001
IL-18	8.6 ± 1.5	^&^25.2 ± 5.5	^&^23.4 ± 2.3	
CCL2	13.0 ± 1.3	^&^142.5 ± 48.8	^&^25.2 ± 2.7	^*∗*^0.0001
CCL5	60.9 ± 8.8	^&^6584.9 ± 658.2	412.7 ± 107.6	^*∗*^0.0001
CCL11	15.5 ± 2.9	^&^157.5 ± 18.5	^&^33.9 ± 3.5	^*∗*^0.0001
CCL27	122.7 ± 9.8	^&^39.3 ± 6.6	^&^65.4 ± 5.2	^*∗*^0.01
CXCL9	126.3 ± 19.1	^&^1844.2 ± 476.7	^&^1331.7 ± 115.8	
CXCL10	49.1 ± 7.2	^&^6741.3 ± 652.5	^&^1058.7 ± 120.4	^*∗*^0.0001
G-CSF	8.1 ± 0.9	^&^50.9 ± 4.7	17.7 ± 1.0	^*∗*^0.0001
G-MCSF	2.6 ± 0.7	^&^474 ± 7.8	^&^6.7 ± 0.8	^*∗*^0.0001
HGF	95.9 ± 13.7	^&^433.8 ± 59.4	^&^283.0 ± 2.4	^*∗*^0.05
IFN-*γ*	32.6 ± 4.4	^&^139.8 ± 32.2	^&^76.4 ± 6.7	^*∗*^0.01
M-CSF	1.5 ± 0.1	11.9 ± 4.9	^&^3.0 ± 0.3	
MIF	145.7 ± 25.9	^&^393.1 ± 71.4	^&^342.1 ± 45.1	
PDGF-b	144.6 ± 24.0	15675.3 ± 11482.3	^&^626.6 ± 73.4	^*∗*^0.05
SCGF	1569.1 ± 246.8	^&^7425.2 ± 1016.7	^&^5874.4 ± 605.3	
TRAIL	15.8 ± 2.5	^&^41.3 ± 6.9	^&^42.2 ± 3.5	
VEGF	15.2 ± 2.5	^&^452.3 ± 67.7	^&^28.2 ± 1.8	^*∗*^0.0001

^&^Significant differences between group and control.
